# Therapeutic Efficacy of Artificial Skin Produced by 3D Bioprinting

**DOI:** 10.3390/ma14185177

**Published:** 2021-09-09

**Authors:** Kwang-Sik Jang, Soon-Jung Park, Jong-Jin Choi, Ha-Na Kim, Kyung-Mi Shim, Mi-Jeong Kim, Il-Ho Jang, Song-Wan Jin, Seong-Soo Kang, Se-Eun Kim, Sung-Hwan Moon

**Affiliations:** 1 Department of Veterinary Surgery, College of Veterinary Medicine and Biomaterial R&BD Center, Chonnam National University, Gwangju 61186, Korea; rhkdtlr0327@nate.com (K.-S.J.); simchung-98@hanmail.net (K.-M.S.); 2 Pangyo Research Center, T&R Biofab Co., Ltd, Seongnam-si 13487, Korea; parksoonjung@gmail.com (S.-J.P.); kimmj@tnrbiofab.com (M.-J.K.); 1hojang@tnrbiofab.com (I.-H.J.); songwan@tnrbiofab.com (S.-W.J.); 3 Tera Science Co., Ltd., Changwon-si 51539, Korea; magicisdream@naver.com; 4 Department of Medicine, Konkuk University School of Medicine, Seoul 05029, Korea; flower21633@gmail.com

**Keywords:** artificial skin, dECM, 3D bioprinting, chimney wound model, mouse

## Abstract

The skin protects the body from external barriers. Certain limitations exist in the development of technologies to rapidly prepare skin substitutes that are therapeutically effective in surgeries involving extensive burns and skin transplantation. Herein, we fabricated a structure similar to the skin layer by using skin-derived decellularized extracellular matrix (dECM) with bioink, keratinocytes, and fibroblasts using 3D-printing technology. The therapeutic effects of the produced skin were analyzed using a chimney model that mimicked the human wound-healing process. The 3D-printed skin substitutes exhibited rapid re-epithelialization and superior tissue regeneration effects compared to the control group. These results are expected to aid the development of technologies that can provide customized skin-replacement tissues produced easily and quickly via 3D-printing technology to patients.

## 1. Introduction

The skin is a complex organ that provides protection, exhibits regulatory functions, and is responsible for communication between the external environment and internal organisms [1]. In general, skin injuries can heal spontaneously [2]. However, the treatments for extensive burns, chronic wounds, and other wounds involving remarkable skin loss are presently limited; moreover, autologous split-thickness skin grafts are still used as a standard treatment [3,4]. Recently, various skin substitutes, such as Integra®, AlloDerm®, Dermagraft®, and Matriderm® have been commercialized; however, most skin substitutes have disadvantages such as relative simplicity of tissue engineering structures, very few therapeutic effects, and immune rejection responses [5,6,7,8]. Therefore, skin substitutes that replace the entire function of the skin and represent a more complex system that integrates the structure of the skin with multiple cellular phenotypes are urgently required. From this perspective, 3D-printing technology, bioink, and artificial skin bioprinting technology that imitates the external skin structure and microenvironment have gained immense attention. It was reported that the artificial skin produced in this way had an excellent effect on wound healing by promoting collagen remodeling and skin regeneration in the wounded area [1,3,9,10]. In order to successfully produce artificial tissues or organs using 3D cell-printing technology, a suitable 3D bioprinter, printing process, and bioink are required [11,12,13]. In particular, the rheological properties of bioinks, such as viscosity and printability, are important, and several materials used as bioinks have been proposed; among these, decellularized extracellular matrix (dECM) is known to provide an environment similar to that of living cells [11,14,15,16,17]. We previously developed a technology for producing dECM-based bioinks that retained collagen, glycosaminoglycan (GAG), and elastin components using porcine skin extracellular matrix, and further devised a technology to produce skin substitutes using 3D-printing technology [11,14]. Herein, we synthesized 3D-printed skin substitutes using porcine-derived dECM and skin constituent cells of keratinocytes and human fibroblasts in a skin defect model. Skin defect models evaluate skin regeneration by an artificial wound such as an incision, excision, or burn. A newly proposed mouse chimney wound model could be a suitable animal experimental model for examining the recovery of skin wounds in that it is similar to that of a human, so in this study, we assessed the therapeutic effects of 3D-printed skin substitutes in an environment mimicking the human wound-healing process [18].

## 2. Materials and Methods

### 2.1. Fabrication of Chimney Structures Using a 3D Printer

The chimney structures were produced using a commercial 3D printer (3DP-310F, Cubicon, Newark, NJ, USA), and were further transplanted into the mouse model. To produce these structures, we used SolidWorks (SolidWorks Corporation, Waltham, MA, USA), a computer-aided design software. The modeled chimney structure was converted from Cubicon’s software into the G-code for final 3D printing. The material used filaments of polylactic acid (PLA) (Cubicon), a biodegradable polymer material. A hollow structure with inner and outer diameters of 6.4 and 9 mm, respectively, and a height of 1 mm was constructed to provide stability to the chimneys. The 3D-printed chimney structures were sterilized overnight with 70% ethanol. A skin dECM-based bioink was printed at 15 °C with a pneumatic pressure of 30–40 kPa in the manufactured chimney (3DX printer, T&R Biofab, Siheung, Korea).

### 2.2. Production of Artificial Skin with Skin dECM and Human Cells

Skin dECM bioink was extracted from porcine skin and produced in the form of a sponge through the decellularized process, and was provided by T&R Biofab (deCelluid, Siheung, Korea). The 5% skin dECM solution was prepared by dissolving in 0.02 M acetic acid for 5 days at 4 °C [11,14]. To adjust the pH of the bioink, a resuspension buffer (RB) comprising NaHCO_3_ (2.2 g), HEPES 200 mM (4.77 g), and 0.05 M NaOH (0.2 g) dissolved in 100 ml of distilled water was used. The prepared skin dECM bioink, RB, and 10× minimum essential medium were mixed on ice at a ratio of 8:1:1 and neutralized to yield a pH of 6.7–7.0. Then, 8 × 10^5^ human skin fibroblasts/mL (Lonza, Walkersville, MD, USA; CC-2511) were further mixed into the neutralized bioink, and residual bubbles were removed by centrifugation at 1200 rpm for 5 min (at 4 °C). The prepared bioink was delivered for the 3D-bioprinting process. After 3D bioprinting, gelation of skin bioink was carried out at 37 °C in an incubator for 40 min. Thereafter, human epidermal keratinocytes (Lonza, Walkersville, MD, USA; 00192627) were plated (at a density of 2.5 × 10^5^ cells/well) over gelated bioink and submerged into keratinocyte growth medium (Lonza, Basel, Switzerland, KBM-Gold 00192151 with Gold SingleQuot 00192152) for 48 h [19,20]. Next, this sample was transferred to the chimney wound model under air–liquid interface culture conditions. The dECM bioink was printed via a 500 um nozzle at 500 mm/min. Rheological analysis and CCK-8 were performed to verify the printability of the bioink and the cell proliferation of the 3D-printed skin bioink, respectively. We conducted rheological analyses of viscoelasticity and viscosity to confirm the properties of the bioink. Viscoelasticity was measured in temperature ranges of: 4 to 15 ℃ for 10 min, 15 ℃ for 10 min, 15 to 37 ℃ for 10 min, and 37 ℃ for 30 min. The printed cell proliferation test was conducted using a CCK-8 (Dojindo Molecular Technologies, Inc., Rockville, MD, USA). The cell proliferation test confirmed the proliferation of printed dermal cells depending on the period of time. It was measured at 450 absorbance in the plate reader (Victor Nivo, PerkinElmer, Inc., Waltham, MA, USA). Only dermis was printed because dermis is a highly viscous material with shearing properties, making it difficult to produce evenly in small areas. On the other hand, in this study, the epidermis was composed of a single layer and produced using suspension to facilitate the air–liquid interface condition after transplantation.

### 2.3. Generation of the Mouse Chimney Wound Model

For the wound-healing study, we used a chimney model mimicking the human wound-healing process [18,21]. This chimney model was established in Balb/c nude mice (Orient Bio Inc., Gyeonggi-do, Korea), as described in a previous study [18]. Briefly, mice were anesthetized with an intraperitoneal injection of 40 μL of a solution containing ketamine (10 mg/kg; Ketamine, Yuhan Co., Seoul, Korea) and xylazine (40 mg/kg; Rompun®, Bayer, Leverkusen, Germany). After disinfecting each mouse by swabbing with alcohol, an excisional wound was made at the middle of the dorsal surface by using a 10 mm biopsy punch (Acuderm Inc., Fort Lauderdale, FL, USA). Thereafter, a chimney and 3D-printed artificial skin were inserted into the excisional wound. Subsequently, the chimney opening was sealed using a transparent film (Opsite, Smith & Nephew, London, UK) to prevent the drying and entry of external substrates.

### 2.4. Histological Analysis (Staining Using Hematoxylin and Eosin (H&E)/Masson′s Trichrome Stain) and Immunohistochemistry Analysis

For the histological analysis, the mice were sacrificed and tissue samples were obtained on days 3, 6, and 12. The samples were fixed in 4% paraformaldehyde, dehydrated using a graded ethanol series, and embedded in paraffin. The samples were obtained at a thickness of 5 mm and stained with H&E to confirm the presence of the epidermis and cell infiltration. Moreover, staining with Masson’s trichrome (MT) stain was performed to assess the presence of collagen index (thickness and maturation) in the wound-regeneration tissues. To evaluate the immune cell expression (macrophages), re-epithelialization, and proliferation, the tissue sections were stained with anti-F4/80 (Thermo Scientific, Waltham, MA, USA, cat no. 14-4801-82), anti-CK14 (cytokeratin14, Abcam, cat no. ab181595), and anti-Ki67 (Thermo Scientific, Waltham, MA, USA, cat no. 11-5698-82) antibodies. The staining signals were developed using an avidin peroxidase system (ABC kit; Vector Laboratories, Burlingame, CA, USA), and hematoxylin (Sigma-Aldrich, St. Louis, MO, USA) was used for counterstaining.

### 2.5. Statistical Analysis

Segmented wound samples were used to measure collagen deposition on the indicated days and quantified by using ImageJ software (version 6.0, National Institutes of Health, Bethesda, MD, USA). Quantitative data are expressed as mean standard error of mean. Statistical analysis was performed with t-tests using SPSS software (SPSS Inc, Chicago, IL, USA). A value of *p* < 0.05, *p* < 0.01, or *p* < 0.001 was considered significant.

## 3. Results and Discussion

### 3.1. Properties of the Bioink and the Cell Proliferation of the 3D-Printed Skin Bioink

**Figure 1:**
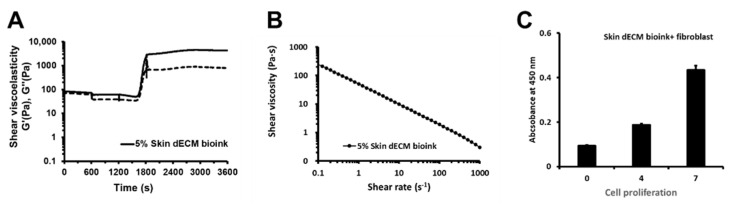
Rheological analysis of the bioink’s printability and cell proliferation of the 3D-printed skin bioink: (**A**) viscosity of the bioink; (**B**) viscoelasticity of the bioink; (**C**) cell proliferation of the 3D-printed skin bioink.

To analyze the properties of the bioink, it tested in a rheological analyzer (Marvern KINEXUS Pro+, Worcestershire, UK) for factors such as viscoelasticity and viscosity (Figure 1A,B). Viscoelasticity was measured in temperature ranges of: 4–15 ℃, 15 ℃, 15–37 ℃, and 37 ℃. results for viscoelasticity showed that our bioink was thermally gelated at 37 ℃, and after gelation, maintained its structure. Viscosity was measured at 37 ℃ with a shear rate (s^−1^) of 0.1–1000. The results for viscosity showed the character of a shear-thinning material that reduced viscosity depending on Pa∙s. The viscosity results showed that our bioink was printable. We conducted the cell proliferation analysis by printing skin bioink mixed with fibroblasts to verify whether 3D printing affected the cell proliferation rate (Figure 1C). The results showed time-dependent cell viability from day 0 to day 7. These results confirmed that cells could stably proliferate in printed structures.

### 3.2. Development of a Skin-Tissue-Manufacturing Technology Using 3D Bioprinting

**Figure 2:**
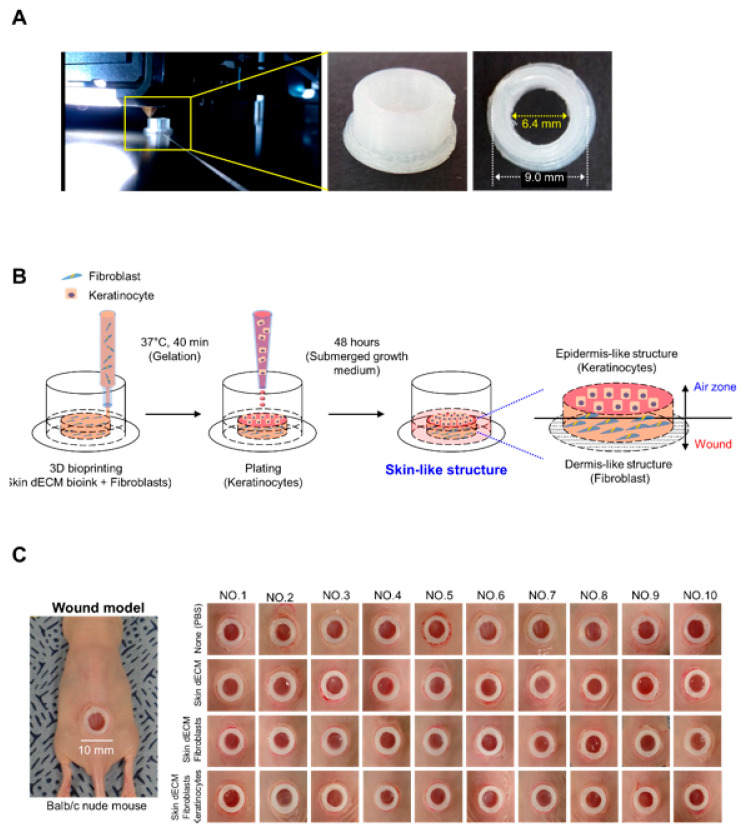
Generation of chimney structures and skin substitutes by 3D printing: (**A**,**B**) fabrication of chimney structures using a 3D printer and preparation of skin substitutes via 3D cell-printing technology; (**C**) confirmation of uniform chimney model production in all experimental groups.

In the present study, we used a chimney wound model to analyze the therapeutic efficacy of 3D-printed skin substitutes in an environment that was highly similar to that of the human body. There are various animal models to evaluate the recovery of skin wound, especially with rodents. However, the mechanism of skin regeneration in rodents differs from that of humans. The chimney wound model can simulate the human wound-healing process in mice; it involves the implantation of a structure called a chimney to prevent wound contraction [18,21]. The conventional chimney wound model uses a nonbiocompatible material known as a microtube. Moreover, we improved the chimney structure using PLA, a noncytotoxic biocompatible component. The chimney structure was manufactured with outer and inner diameters of 9.0 and 6.4 mm, respectively (Figure 2A and Video S1 in the Supplementary Materials). Porcine skin–dECM bioink and fibroblasts were mixed in the interior of the fabricated chimney structures and applied uniformly to a height of 8 mm by 3D cell-printing technology; next, keratinocytes were plated to prepare skin substitutes. The skin substitutes were transplanted into mice after 48 h of incubation such that the keratinocytes contacted the air zone and fibroblasts contacted the wound site (Figure 2B). The 3D-printing technology was able to quickly produce a uniform skin tissue, and the produced skin tissue was implanted onto the dorsal region of the mouse. To produce the chimney model, an excisional wound (10 mm in size) was made on the dorsal region of the anesthetized Balb/c nude mice using a biopsy punch. The chimney structure and skin substitute were inserted into the excisional wound, and were fixed using Dermabond (Ethicon, Inc., Somerville, NJ, USA) to prepare the chimney model. In order to analyze the therapeutic efficacy of the skin substitutes, we used the untreated mice as the control group; 10 mice each in the skin–dECM, skin–dECM + fibroblasts, and skin–dECM + fibroblasts + keratinocytes experimental groups were compared. It was confirmed that all 40 animal models were uniformly established (Figure 2C).

### 3.3. Optimization and Experiment Strategy of Chimney Wound Model

**Figure 3:**
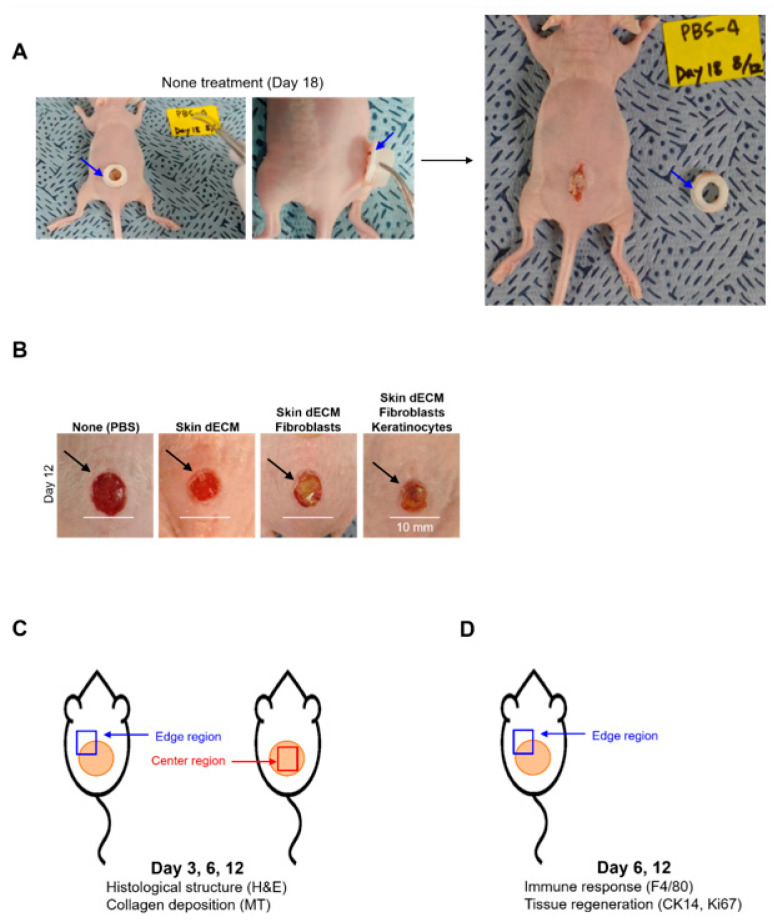
Evaluation of the usefulness of the 3D-printed chimney structures: (**A**) inspection of the maintenance of the 3D-printed chimney structures on day 18; (**B**) inhibition of wound contraction following the implantation of the chimney structures at 12 days; (**C**,**D**) analysis of the wound samples.

Prior to evaluating the therapeutic efficacy of the skin substitutes, maintenance of the produced chimney structures was confirmed. The fabricated chimney structures were composed of PLA; it was confirmed that the chimney structures were maintained and did not dismantle. Nevertheless, 18 days after the implantation of the untreated (PBS, phosphate buffer saline) chimney structures, the chimney structures were separated from the mice, and wound contraction was observed due to the deviation of these structures (Figure 3 and Video S2 in the Supplementary Materials). This can be explained by the weakening of adhesion, as the natural wound of the mice in the region under the PLA-containing chimney structure was healed (Figure 3A, blue arrows). Therefore, we selected 12 days as the time point at which results different from those in the mice from the control group were obtained; when the wounds from which the chimney structures were removed were visually confirmed after 12 days, it was confirmed that no natural healing was observed in the mice from the control group. During this period, the chimney structures inhibited the wound contraction in mice from the control group and the size of the first resected wound was maintained, whereas inflammatory reactions were observed visually in the mice from the experimental group (Figure 3B, black arrows). The experiment was conducted for a maximum of 12 days, and the histological changes in the wound and the degree of collagen deposition were analyzed by dividing the center region and the edges on days 3, 6, and 12. Furthermore, the immune response and tissue regeneration were analyzed based on cell transplantation, which was performed on the edges of the wound on days 6 and 12. Although we verified the therapeutic effects of the skin structures in mice, these results may represent the exact therapeutic efficacy of the 3D-printed artificial skin structures in humans, because our analysis was performed in an environment that was highly similar to that associated with natural human wound healing.

### 3.4. Histological Analyses of Wound Tissues in the Chimney Model

**Figure 4:**
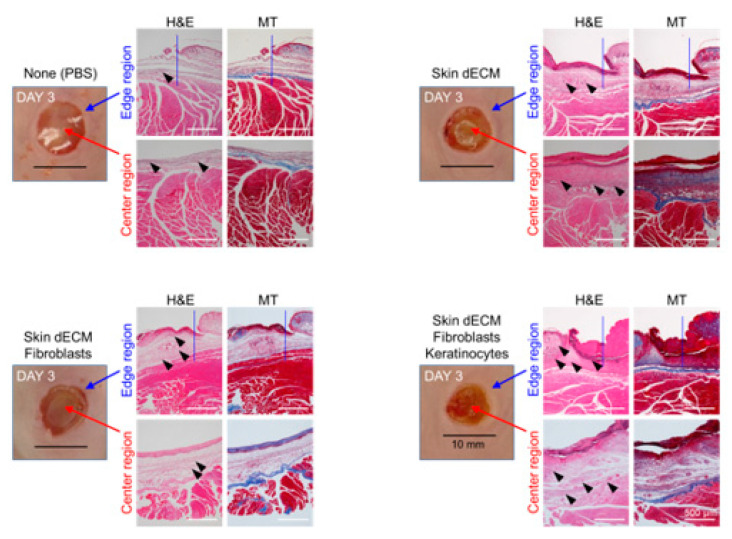
Histological analysis of cell infiltration and collagen deposition. Tissues stained with hematoxylin and eosin, and Masson’s trichrome stain, were analyzed at various stages on day 3. The images show stained tissue samples depicting the infiltration of cells into the dermal layer (black arrow heads) and fibrosis and collagen deposition at the site of damage (yellow line).

**Figure 5:**
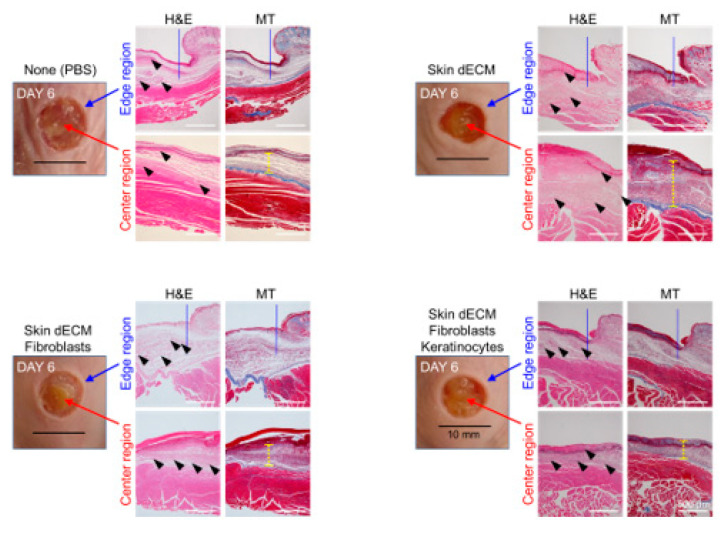
Histological analysis of cell infiltration and collagen deposition. Tissue samples stained with hematoxylin and eosin, and Masson’s trichrome stain, were analyzed at various stages on day 6. The images show stained tissue samples depicting the infiltration of cells into the dermal layer (black arrow heads) and fibrosis and collagen deposition at the site of damage (yellow line).

**Figure 6:**
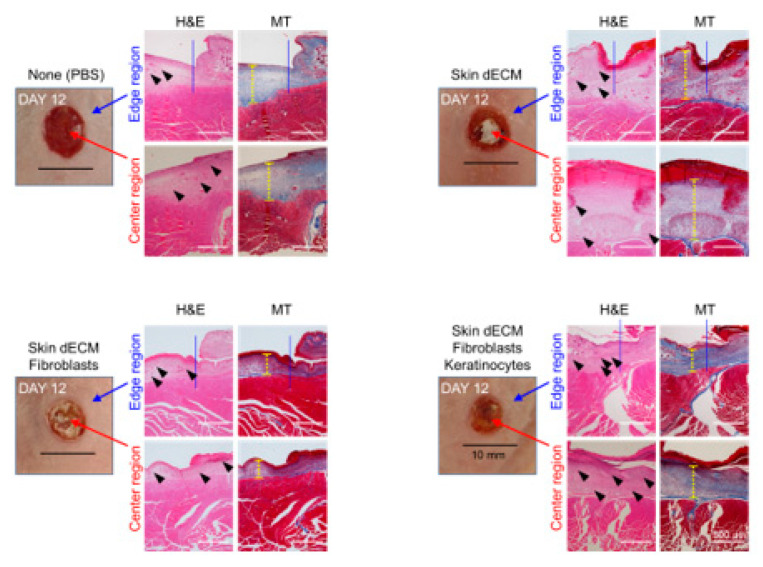
Histological analysis of cell infiltration and collagen deposition. Tissue samples stained with hematoxylin and eosin, and Masson’s trichrome stain, were analyzed at various stages on day 12. The images show stained tissue samples depicting the infiltration of cells into the dermal layer (black arrow heads) and fibrosis and collagen deposition at the site of damage (yellow line).

**Figure 7:**
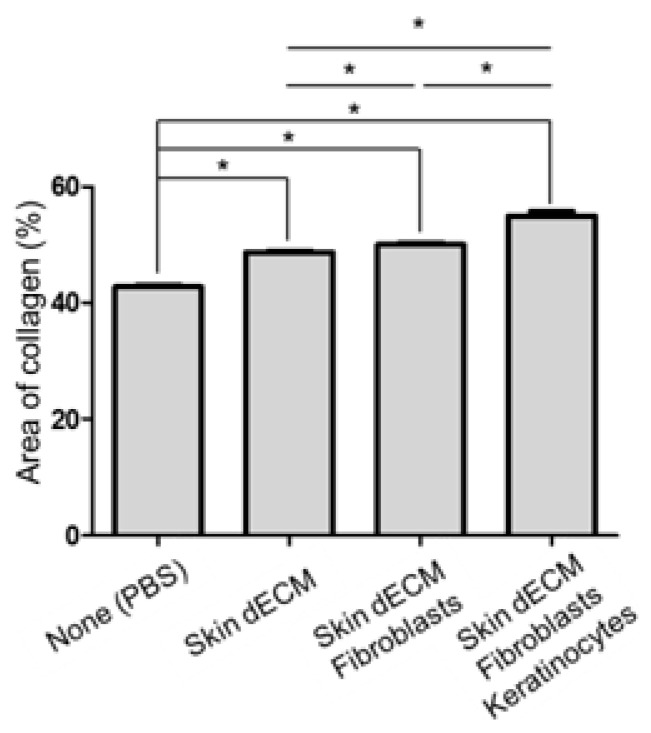
Quantification of area of collagen layer (%) in the histological analysis. Data are presented as the mean ± standard error of mean (*n* = 3 per groups) and analyzed using a Student’s *t*-test (* *p* < 0.05).

Tissue samples were collected at various stages of the healing process on days 3, 6, and 12 to evaluate the treatment effectiveness at the histological level. The analysis of the wound sites and collagen deposition were assessed by H&E and MT staining (wound edge/wound center); further, the immune responses and re-epithelialization were assessed by F4/80, CK14, and Ki67 staining (wound edges) (Figure 3C,D). On day 3, H&E staining indicated that mice from all the dECM-transplanted groups showed faster cell infiltration into the wound site than in case of those from the control group (Figure 4, black arrow heads). These cells appeared to be major cells associated with the wound-healing process, such as inflammatory cells and fibroblasts. Most of the infiltrating cells were considered as inflammatory cells, particularly because day 3 represented the early stage of the wound [22]. Moreover, previous studies have reported that porcine-derived dECM did not induce an immune response. This suggested that the dECM induced cell infiltration, leading to a faster inflammatory phase [3,12]. In addition, the invasion of these cells was observed continuously on days 6 and 12 (Figure 5 and Figure 6, black arrow heads). Masson’s trichrome staining confirmed the severe progression of fibrosis in the center and edges of the wound tissues on day 3 (Figure 4). Even on day 6, residual cells were not observed in the stained area in mice from the PBS control group; however, a high degree of cell infiltration was observed in the mice from the group treated only with dECM. Nevertheless, a high degree of cell infiltration was observed within the wound tissues in the mice that received dECM transplantation along with the skin cells. This cell infiltration was distinct in the center of the wound tissues (Figure 5, yellow line). Collagen deposition could be evaluated by tissue analysis at day 12. In the PBS-treated control group, fibrosis was observed at both the center and edges of the wound tissues, showing a high distribution. In the group treated only with dECM, collagen deposition was partially observed in the edges; on the other hand, a wide distribution of cell infiltration was observed in the center of the wound tissues, but collagen deposition was not observed. Nevertheless, in the mice with transplanted cells, collagen deposition was observed in both the center and edges of the wound tissues; in particular, clear deposition of mature collagen was observed in the mice that were treated with keratinocytes (Figure 6, yellow line). In addition, consistent with our observation, quantification confirmed that the number of area of collagen (%) was highest in the skin–dECM + fibroblasts + keratinocytes group (Figure 7) [23]. From these results, we found that when the fibroblasts and keratinocytes were transplanted, the problem caused by the inflammatory reaction was not serious; furthermore, the mice that were treated with these two cell types showed effective collagen deposition in the wound tissues. Therefore, we expect that the 3D-printed artificial skin structure that was proposed herein can be used as a new alternative for existing skin grafts.

### 3.5. Therapeutic Effects of the 3D-Printed Artificial Skin

**Figure 8:**
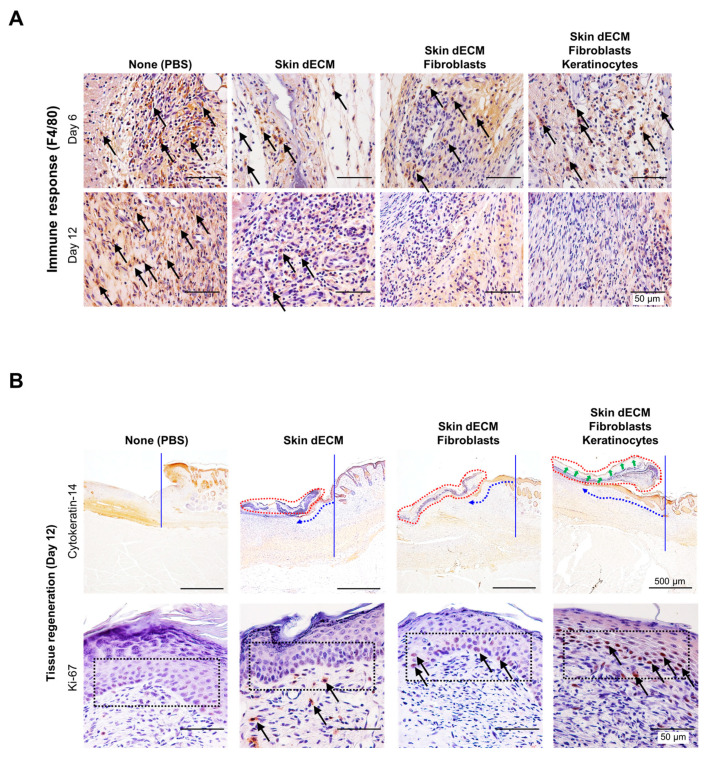
Histological analysis of the inflammatory process and re-epithelialization: (**A**) detection of F4/80 in macrophages within the tissue sections on days 6 and 12 (black arrows) of the inflammatory process; (**B**) detection of CK14 and Ki-67 expression to confirm re-epithelialization (blue arrows) and keratinocyte proliferation (black arrows), respectively.

To further investigate wound regeneration and re-epithelialization associated with immune responses, we performed immunohistochemical analyses for detecting the expression of the markers CK14, F4/80, and Ki67 on days 6 and 12. Staining for F4/80 indicated the recruitment of macrophages to the wound site. The degree of macrophage recruitment decreased gradually in all groups on day 6, and in the dECM group on day 12. Specifically, macrophages disappeared from the cell-transplanted group on day 12. However, in the nontreated group, it was confirmed that the inflammatory response by macrophages was continuously maintained until 12 days (Figure 8A, black arrows). These results suggested that skin substitutes containing skin cells displayed a faster rate of immune cell reaction than only-dECM-based skin substitutes, and that the inflammatory phase associated with the healing process proceeded faster in the case of the former [22]. Epidermal regeneration was confirmed via the staining of cytokeratin 14 and Ki67. The keratinocyte marker Ki67 was significantly expressed in samples from all the dECM-transplanted groups, compared to that in the control group. The mice from all the dECM-transplanted groups showed epidermal keratinocyte migration from the wound edges to the center of the wound sites, whereas the mice from the control group did not show keratinocyte migration (Figure 8B, blue arrow). Moreover, the fastest re-epithelialization was seen in the mice from the group treated with keratinocytes, indicating that the material secreted from the transplanted cells influenced the regeneration of cells in the host tissues (Figure 8B, black arrow) [24]. As a result of the division of the keratinocytes involved in re-epithelialization, which was assessed by Ki-67-staining analysis, the mice in the control group did not show cell division in the tissues adjacent to the basal layer of the epidermis; however, dividing cells were identified in mice from all the dECM-treated groups, and the highest degree of cell division was observed in case of the group treated with keratinocytes (Figure 8B, black arrows). Although tissue regeneration and re-epithelialization occurred rapidly in mice treated with keratinocytes and fibroblasts, wound healing was more effective in case of mice from the only-dECM-treated group than in those from the control group. However, from these results, we could not confirm that the cells in the artificial skin survived until analysis, but we could confirm that the cells engrafted and assisted in the wound-healing process.

These results revealed two main properties of porcine-derived dECM. First, the artificial skin in keratinocytes and fibroblasts promotes re-epithelialization by synthesizing abundant fibronectin in the dermal-epidermal basement membrane region [25]. Second, porcine-derived dECM does not induce an inflammatory reaction and contains components such as collagen, GAG, and elastin; hence, it can provide important biochemical signals for cellular activities such as cell adhesion, proliferation, and differentiation [26,27]. Third, porcine-derived dECM contains 24 types of residual growth factors, such as basic fibroblast growth factor, fibroblast growth factor, and keratocyte growth factor, which can accelerate tissue regeneration [14].

As a result, porcine-derived dECM exerts its own therapeutic effects without the aid of surrounding tissues. In this study, porcine-derived dECM, which is effective for tissue regeneration, was used as a bioink, and artificial skin tissues were produced using 3D-printing technology; skin substitutes containing fibroblasts and keratinocytes were prepared. Furthermore, the therapeutic efficacy of the 3D-printed skin substitutes was confirmed using a mouse chimney wound model that mimicked the normal wound-healing process in the human body. Therefore, we believe that 3D-printed skin tissue cells can serve as a new alternative to skin grafts used to treat burns and wounds, the current supply of which is limited.

## 4. Conclusions

In this study, 3D-printed skin using a bioink based on dECM derived from porcine skin with human dermal fibroblasts and keratinocytes was produced and evaluated for its skin wound-healing effect using a mouse chimney wound model. The 3D printed skin, with a structure similar to the skin layer, exhibited rapid re-epithelialization and superior tissue regeneration in the histological and immunohistochemical analyses of the animal experiment. We believe that clinical validation should be carried out in chronic skin wound models or in larger skin defect models in order to be applied directly to clinical care in the future. However, based on the results of this study, it is expected that 3D-printed artificial skin may be used as a skin substitute for treatment of burns and autologous skin transplantation.

## Data Availability

Data sharing not applicable.

## Supplementary Materials

The following are available online at https://www.mdpi.com/article/10.3390/ma14185177/s1, Video S1: Supplementary Video 1 is the video file for Figure 2A. Video S2: Supplementary Video 2 is the video file for Figure 3.
